# Identifying Characteristic Fire Properties with Stationary and Non-Stationary Fire Alarm Systems

**DOI:** 10.3390/s24092772

**Published:** 2024-04-26

**Authors:** Michał Wiśnios, Sebastian Tatko, Michał Mazur, Jacek Paś, Jarosław Mateusz Łukasiak, Tomasz Klimczak

**Affiliations:** 1 Division of Electronic Systems Exploitations, Institute of Electronic Systems, Faculty of Electronics, Military University of Technology, 2 Gen. S. Kaliski St., 00-908 Warsaw, Poland; michal.wisnios@wat.edu.pl (M.W.); jacek.pas@wat.edu.pl (J.P.); jaroslaw.lukasiak@wat.edu.pl (J.M.Ł.); 2 Doctoral School, Faculty of Electronics, Military University of Technology, 2 Gen. S. Kaliski St., 00-908 Warsaw, Poland; sebastian.tatko@wat.edu.pl; 3 Department of Building Safety, Fire University, 52/54 J. Słowackiego St., 01-629 Warsaw, Poland; tklimczak@apoz.edu.pl

**Keywords:** fire detectors, non-stationary fire alarm system, false alarm

## Abstract

The article reviews issues associated with the operation of stationary and non-stationary electronic fire alarm systems (FASs). These systems are employed for the fire protection of selected buildings (stationary) or to monitor vast areas, e.g., forests, airports, logistics hubs, etc. (non-stationary). An FAS is operated under various environmental conditions, indoor and outdoor, favourable or unfavourable to the operation process. Therefore, an FAS has to exhibit a reliable structure in terms of power supply and operation. To this end, the paper discusses a representative FAS monitoring a facility and presents basic tactical and technical assumptions for a non-stationary system. The authors reviewed fire detection methods in terms of fire characteristic values (FCVs) impacting detector sensors. Another part of the article focuses on false alarm causes. Assumptions behind the use of unmanned aerial vehicles (UAVs) with visible-range cameras (e.g., Aviotec) and thermal imaging were presented for non-stationary FASs. The FAS operation process model was defined and a computer simulation related to its operation was conducted. Analysing the FAS operation process in the form of models and graphs, and the conducted computer simulation enabled conclusions to be drawn. They may be applied for the design, ongoing maintenance and operation of an FAS. As part of the paper, the authors conducted a reliability analysis of a selected FAS based on the original performance tests of an actual system in operation. They formulated basic technical and tactical requirements applicable to stationary and mobile FASs detecting the so-called vast fires.

## 1. Introduction

Electronic security systems (ESSs) employed in buildings or within a vast area are temporarily tasked with ensuring the safety of both animate (e.g., humans, animals, etc.) and inanimate (e.g., natural environment, forests, soil and water) matter. One of the most important security systems applied within human surroundings (near and far) is the FAS. The operation, organization and maintenance of this system within a given space utilized by humans has been thoroughly defined and set out in acts and legal regulations applicable in specific countries, e.g., in Poland, pursuant to Dz.U. 109, item 719 [1]. The acceptance and commissioning of a given building requires, in the case of an FAS used within a given space, verifying its correct functioning in real fire hazard scenarios simulated by authorized persons. In Poland these are officers of the State Fire Brigade (SFB). The verification also covers design diagrams, connections, detection line and circuit routing within the protected buildings and alarm and failure signal transmission channel latency and availability, as well as individual elements, devices and modules of the entire FAS [2,3]. Pursuant to Annex 1 to the Regulation of the European Parliament No. 305/2011 dated 9 March 2011, all buildings treated as a whole and their individual sections (e.g., windows, doors, sensors, building materials, etc.) must be suitable for use according to their intended purpose. The health and safety of people and persons in contact with them throughout the entire service life of these technical facilities must be particularly considered in this case [1,3]. The applied FAS elements, devices and modules must satisfy, among other requirements, specific fire safety requirements. Pursuant to the applicable Annex No. 4 to this EU regulation, fixed extinguishing devices (FEDs) and fire detection (detectors, sensors, etc.) and signalling (Fire Alarm Control Panels—FACP) products, acoustic and optical devices (AOD) or Manual Call Points (MCP), etc., were classified into the code 10 group. All FAS components were classified as building products, extremely important from the perspective of fire safety in the light of the implemented functions (such as, e.g., ceiling, joist, windows, doors, stair treads and risers, internal walls, etc.) [4,5]. Therefore, all FAS components, modules, elements, devices, cables, supports and mechanical connectors must be awarded a specific test certificate approving their use within the entire country. Such a certificate is issued in Poland by the CNBOP (Scientific and Research Centre for Fire Protection) of the National Research Institute in Józefów [6,7,8]. The FAS fire surveillance of many individual buildings grouped within a vast area means the application of stationary security systems [2,3]. However, scattered or non-stationary FASs are employed in the case of fire detection in facilities located within a vast area, such as warehouses, logistics and navy bases, airport areas, railway stations and adjacent land, tracks and turnouts, marshalling yards, aircraft repair hangars and military or police units with adjacent training grounds, especially for identifying fire characteristic values (FCV) [9,10,11]. All of the aforementioned facilities are classified as the so-called state critical infrastructure (SCI) [12,13,14]. Depending on the technical structure, intended purpose, control and internal communication method, as well as facility, building or vast area protection, FASs can be divided into three major groups:

- A focused FAS intended for the fire protection of small-sized facilities or buildings, with their area expressed in [m^3^]. FCV detectors (sensors), as well as MOP or AOD, are hooked up to all detection circuits and loops. A circuit always starts and ends in an FACP. It is a stationary system that detects a fire hazard at its installation location [15,16,17]. Such an FAS type was considered within this study.
- A scattered FAS is employed for fire surveillance over a vast area, in several or a dozen and more buildings, within areas and facilities classified as SCI. Depending on the monitored area, an FAS has from two to even a dozen or so (and even several hundred in certain cases) FACPs, properly communicated in terms of telematics, with master and slave control units always present. Such a system can also be classified as stationary, since it detects fire hazards at the installation locations of its sensors and MCPs [18,19,20].
- A mixed FAS consists of the two groups referred to above. A focused FAS monitors the most important facilities within a given area, e.g., an administration building, hangars or fuel depots, while a scattered FAS monitors all other buildings and the terrain within the protected area. It is a stationary system that detects a fire hazard at its installation location [2,21,22].

All three FAS groups described earlier are stationary fire protection systems. Stationary meaning at the place of their installation, operation and maintenance. The major FAS output waveforms are the signals generated by the FACP, namely, alarm, failure and monitoring. An FAS always has the highest priority within the entire system. It is directly transmitted via two independent telecommunications channels to the SFB and the alarm receiving centre (ARC) [23,24]. An alarm signal also automatically interrupts the system monitoring process and the failure signal transmission only to an ARC (a remote service location). The basic FAS operation type is monitoring, which means FCV detection by all sensors, detectors and people within a protected area, in which case an MCP is activated [25,26].

In general, non-stationary FASs are used for detection and prediction (identification in terms of, e.g., fire hazard propagation direction, exposed area size determination, smoke propagation direction, fire and smoke column height, etc.). They also include ongoing surveillance, e.g., officers responsible for the implementation of the fire-fighting process viewing the spread of a fire over a vast area on an LCD display [27,28]. The technical and operational aspects of non-stationary systems are currently developed and expanded with new functions that enable applying all available information to contain a fire source as soon as possible. Fire hazard detection platforms are installed onboard unmanned aerial vehicles (UAVs) and drones are equipped with various sensors to also detect chemical or biological contamination (military applications). These systems are increasingly employed in other situations critical to a country, e.g., floods, heavy rainfall or hail. This includes determining a risk area, landslide, river surges after heavy rainfall, e.g., in the mountains, river catchment storage reservoir fill level and forest stand damage extent after, e.g., a tornado, hurricane or storm, etc.

**Figure 1:**
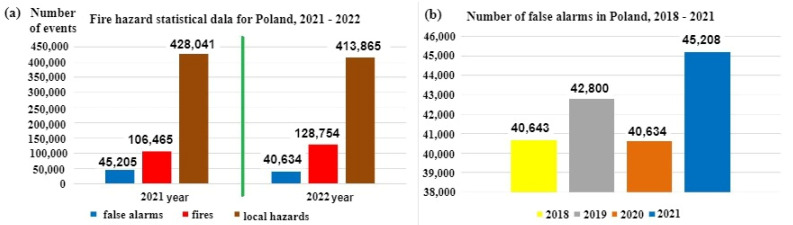
Statistical data related to fires, false alarms and local hazards in Poland: (**a**) general fire characteristics, including false alarms, and (**b**) false alarms in the years 2018–2021.

A so-called false alarm (FA) or a false calm (FC)—a decision developed in an FACP based on input signals from detectors, sensors and MCP—is a very important FAS-related issue [6,17]. Therefore, these systems employ various modern technical, as well as organizational, solutions to minimize the P_FA_ and P_FS_ probabilities [2,6]. The appearance of such signals in an FAS and their transmission to SFB or ARC means, in the case of fire-monitored areas, the suspension of, e.g., land, sea or air traffic, calling emergency services, announcing evacuation and redirecting means of transport to other ports, airports or railway stations [2,15,29]. Such a course of events is associated with a fire hazard, meaning, especially within transport areas, tangible economic losses (expressed in, e.g., dollars) or intangible, incalculable damage (e.g., image-related) [4,17]. Selected SFB statistical data regarding the fire hazards, false alarms and local hazards are shown in Figure 1a,b.

Figure 1b shows data concerning false alarms for the years 2018–2021 in Poland [29]. Based on statistical data from the years 2018–2021, the number of false alarms has been steadily increasing, as shown in Figure 1b, with the share of false alarms in the total number of fires in 2021 reaching 42.463%. This is a very high result and therefore, FAS designers, as well as manufacturing plants and research centres dealing with FCV identification issues, suggest, already at an initial fire stage, ever newer technical solutions aimed at reducing the values of the P_FA_ and P_FAS_ probabilities.

**Figure 2:**
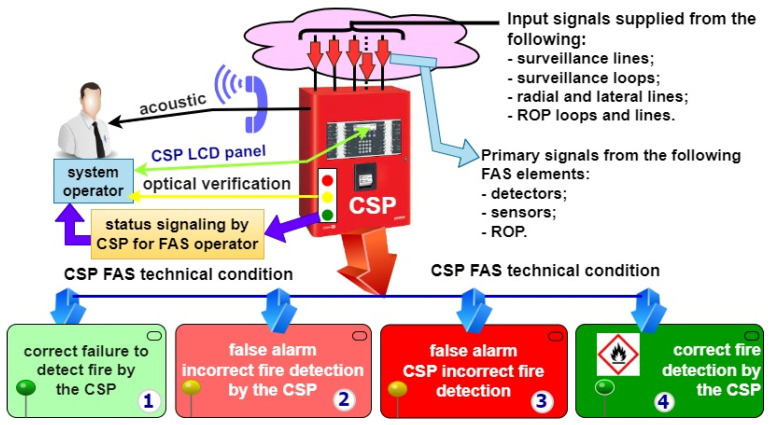
FACP processing of primary fire hazard signals based on standardized electronic signals from detection loops and circuits within a protected area.

Figure 2 is a simplified illustration of the FACP role in processing the primary signals related to a fire hazard within the protected area. The fire hazard information in the form of standardized electrical signals is transmitted directly from detection loops and circuits, radially and laterally, as well as from MCPs, to the FACP. A fundamental function implemented by the FACP within the entire FAS is the decision and signalling (simultaneously for technical states) of the entire system in the form of acoustic, optical and visual information on the control panel [30,31]. The information is sent to a local FAS operator supervising the operation process, and also, to the alarm and failure signal transmission device (AFSTD) simultaneously and automatically transmits the information via two independent telecommunications channels to the ARC and SFB. Based on input signals, an FACP is able to develop four technical states sent to the SFB and ARC. These are the following technical states [2,6,8]:

- The correct non-detection of a fire by an FACP (state No. 1)—correct FACP response to primary signals received from detection loops and circuits—Figure 2;
- A false alarm, the incorrect non-detection of a fire by an FACP (state No. 2)—incorrect FACP response to primary signals received from detection loops and circuit;
- A false calm, the incorrect non-detection of a fire by an FACP (state No. 3)—incorrect FACP response to primary signals received from detection loops and circuits;
- The correct detection of a fire by an FACP (state No. 4)—correct FACP response to primary signals received from detection loops and circuits—Figure 2.

**Figure 3:**
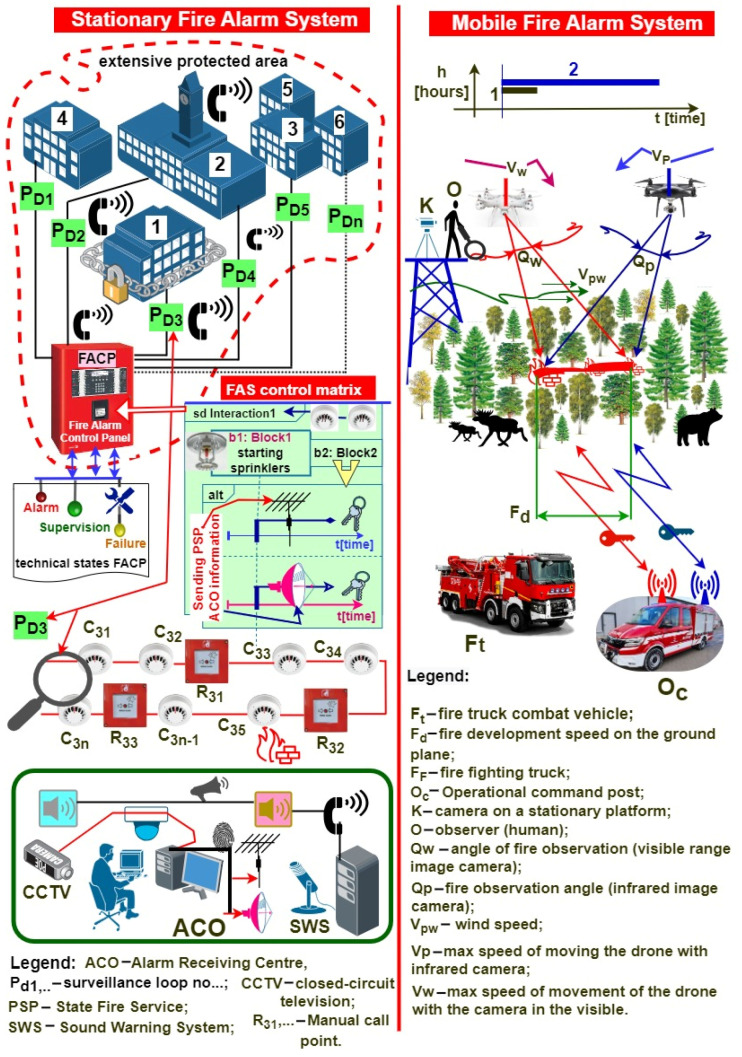
Processing fire hazard primary information in stationary and mobile FAS; basic tactical and technical requirements specified for selected systems.

Figure 3 is a simplified presentation of the method for processing primary information on a fire hazard, and the basic tactical and technical requirements for stationary and non-stationary FAS. These systems exhibit one fundamental difference in terms of intended purpose and combating fire hazards: a mobile FAS only detects a fire hazard within a limited volume, while an FCV sensor platform on a UAV does so over a vast area. A mobile FAS is able to trigger FEDs and GSSs (Gas Suppression Systems), starting upon fire hazard detection and FSB arrival (fire-fighting coordinator) [32,33]. The premature activation of an FED and GSS by an FAS means limiting tangible (calculable) and intangible (air pollution) damage caused by a fire spreading in a building or area, e.g., forest, airport, etc., Figure 3 [2,34].

The roles of stationary and non-stationary FASs in the fire extinguishing process differ. However, the very detection of a fire hazard itself is often a factor that already determines how to make correct decisions regarding fire-fighting at the initial stage of the operations, i.e., applied operational measures, the number of dispatched fire-fighters and fire engines, launching local or area evacuation, etc. [2,4,35]. In general, these are fire-related operational factors and are always individually matched to a given fire type. A stationary FAS implements the fire reconnaissance of a vast area via FACP, using detectors and sensors hooked-up to detection loops P_D1_, P_D2_, P_D3_, …, P_Dn_. Individual detection loops implement the fire-monitoring of buildings No. 1–6. A so-called control matrix is developed for a specific fire hazard to properly and automatically organize the fire-fighting process and trigger FEDs and GSSs. It takes into account all logical and temporal relationships, dependency functions, etc. that have to occur in a specific sequence to trigger specified fire-fighting procedures [8,36]. The control matrix means triggering, e.g., FED sprinklers and transmitting hazard information by an AFSTD to an ARC and the SFB. Figure 3 shows a simplified ARC using other ESS–VSS (Video Surveillance Systems) and AWS (Audio Warning Systems). Only the AWS is integrated within an FAS, while other ESSs only apply fire hazard information. Fire hazard information is broadcast by speakers in protected buildings and within a vast area in an ARC, as illustrated in Figure 3. All telecommunications information incoming/outgoing to/from the FAS and ARC is encrypted [2,6,36]. The primary task of a mobile (non-stationary) FAS located onboard a UAV platform is identifying, supervising and predicting fire spread within vast areas, in this case, as illustrated in Figure 3, a forest on a military training ground, and an airport. Two moving drones, UAVs, (or potentially one or a swarm) carry different sensors for detecting FCVs such as smoke and flames. Identification is also based on two cameras, for detecting images in the visible band and infrared electromagnetic waves. Observation platforms and UAVs exhibit specific technical parameters that determine the extinguishing operation duration:

- Technical: e.g., maximum forward speed V_W_ and V_P_; the permissible observation width of a camera optical system Q_W_ and Q_P_; maximum UAV flight time on a battery bank for a propulsion; UAV control signal transmitters and receivers, i.e., frequency range, band, power, employed interference-resistant modulations, power consumption at different functioning modes (transmitting, receiving, standby, detection, and alarm), etc. [2,4,20,37].
- Tactical: platform and drone dimensions, weight, construction material resistance to shock, overloads, impacts, etc., UAV deployment and retraction time, UAV time to take-off from receiving a hazard signal, permissible UAV flight range under fire search, detection and monitoring conditions, the resistance of transceiver signals to interference, etc. [2,6].

Fire hazard identification using UAVs is still currently under technical development. It is common for stationary fire reconnaissance systems to use cameras and an operator using binoculars, or for another means of communication to be employed (Figure 3). Such fire reconnaissance is inefficient, since it requires incurring specific financial outlay and confirming FCVs for a given area, most usually by an operator responsible for a given forest district (area) [2,9,38]. Time is always the decisive parameter in fire-fighting [2,6,9]. The fire spread information-prediction is sent from the UAV via radio to a fire-fighting command vehicle (O_c_) and an SFB fire engine (F_t_) to properly utilize available human, equipment and fire-fighting resources within the conducted fire-fighting operation. Fire spreading within such an area is also a threat to the natural environment, including the flora, fauna and forest animals located within the exposed area [2,6,39]. Therefore, transmitting specific warning messages (signals) by the UAV, for animals in particular, would be a good solution aimed at avoiding losses. The introduction regarding mobile FASs presents basic requirements, and Figure 3 reviews only basic signals and relationships, for the sake of the figure’s clarity [3,11,40,41]. The proper functioning of two FASs also requires an adequate organization of power supply systems—both primary and backup. The cooperation between stationary and non-stationary FASs is particularly important within the so-called vast fire-monitored areas. These facilities are often part of the so-called state critical infrastructure, e.g., airfields, naval or fuel bases and railway areas with stations and depots. In such a case, fire surveillance at a significant distance from specific structures could be conducted by a non-stationary FAS, and hazard information could be transmitted by a stationary system to the fire brigade. Such cooperation enables the appropriate allocation of extinguishing measures, as well as human and technical resources within a fire-hazard area.

This research paper was organized in the following manner to discuss and present the entire issue related to stationary and non-stationary FASs, and issues associated with FCV identification by these systems, and the operational reliability of selected systems. The first chapter constitutes an overall introduction to the issue of employing stationary and non-stationary FASs, and discusses fundamental issues related to a false alarm. The following section is a critical review of source literature on the issue of stationary and non-stationary FASs, as well as FCVs applied to identify a fire phenomenon. The third chapter focuses on FCVs employed to identify a fire phenomenon, selected FAS structural models and issues associated with a false alarm. This is followed by deliberations related to the use of a non-stationary FAS to detect hazards—the implementation of an observation platform for FCV detection. The penultimate chapter of the paper focuses on a computer simulation developed for the selected FAS reliability model. The last section includes basic tactical and technical assumptions related to a UAV platform. The entire elaboration is summarized with conclusions and references concerning the issues addressed herein. Modern video-based flame and smoke detection systems involving cameras operating within different observation bands (e.g., visible, infrared or ultraviolet) find a growing number of applications as sensors (detectors) detecting a fire hazard in facilities monitored by FASs. However, the application of such state-of-the-art solutions requires equipment certification, since, pursuant to the EU Regulation No. 305/2011—dated 9 March 2011, CPR, all FAS elements shall be treated as building materials. The certification process covering such devices is currently being studied in Poland, and therefore, cameras cannot be operated under FAS structures for the time being. However, employing modern technology in cameras that use, e.g., smart artificial intelligence algorithms, which at an early learning stage (validation) analyse vast amounts of data, such as photographs, video footage, etc., to implement the so-called deep learning processes, will enforce utilizing cameras as part of FAS. These algorithms, when embedded in cameras, additionally supported by high-quality images and, e.g., an infrared radiator, are able to very quickly (within several seconds) and reliably distinguish events—a false fire phenomenon or a fire development initial stage. Owing to novel solutions in cameras and the application of highly efficient processors to process primary signals, fire detection can be conducted in rooms with a very polluted environment, and also with poor visibility, including at night. Previous research by the authors focused on the operational reliability issues associated with FASs with varying technical structures. Acquiring information on failures or the repair process of actual functioning FASs is rather difficult, since their users or manufacturers do not provide such data. However, the FAS functional reliability in the case of conducted tests and computer simulations is at a very high level. Also, the operational tests conducted by the authors of the paper, and focusing on the power supply reliability of operating FASs, confirmed that the applied technical solutions satisfied the technical requirements arising from, e.g., standards.

## 2. Literature Review

When operated, complex and integrated ESSs require the continuous, real-time implementation of a diagnostic process focusing on the technical states of all elements, devices and modules hooked up to an ACP or FACP. In the case of an ESS, it is the alarm control panel that is the device responsible for the entire diagnostic process [4,15,42]. In most cases, an alarm control panel has an additional microprocessor responsible for the diagnostic process only. FASs are diagnosed concurrently with the operating signals from detectors and sensors, while the implementation time and duration of this process depends on the service group [6,17,43]. Information on the diagnostic process is displayed continuously on an FACP LCD screen (Figure 3). In addition, it is also transmitted to an ARC, which supervises system operation within facilities or a vast area. Damage information sent to the ARC enables the immediate undertaking of a recovery process, since the message on an unfitness contains the element number, its location and the failure time for this case [4,44,45]. A service team with an on-site spare parts storage is able to immediately commence a repair. The service and maintenance personnel for FASs located in SCI facilities are located at the system’s operation site. The operation process monitoring team should also have information on the impact of a given unfitness on the entire security system [16,46,47]. Can the occurrence of a given unfitness type cause critical damage? Are there any methods that can be implemented prior to the arrival of the service team ensuring an adequate safety level? Is the occurring unfitness classified as so-called ‘safe’ when applying, e.g., redundant elements or the fail-safe principle? [4,6,46]. In addition to local information on the FAS technical state, the messages are always transmitted via two independent telecommunications channels to relevant services—ARC and SFB [6,47,48]. The interferences occurring within the natural environment should not impact the signal transmission process reliability; this means, e.g., the application of an appropriate transmitter frequency and digital modulations [17,49].

Currently implemented FAS diagnostic processes do not include diagnostic information on redundant elements not directly involved in the functioning of the system. The source literature on the FAS operation process also lacks information on developed forecasts regarding changes in the system reliability R(t) in the case of specific detection loops of circuits becoming unfit [4,16,49]. In the development of an operation process model for a specific system and in conducting the FAS operation process computer simulation, the authors suggest employing an ongoing assessment of the unfitness impact on the R(t) reliability of the entire system [6,17,50]. Output signals carrying damage information that is generated in an FACP do not develop a forecast on the impact of such unfitness on the operation process, let alone fire safety within the protected building [4,6,17,47].

An issue that just cannot be overlooked, particularly in terms of the FAS operation process, is related to the broadly understood interference in the natural environment: mechanical (e.g., vibrations and oscillations of building walls), electromagnetic throughout the entire frequency range, i.e., low-frequency, radiated or high-frequency, radiated interference [4,15,51,52] and temperature-, humidity- and pressure-related interference associated with the change in environmental conditions (e.g., directly affecting a change in the ionization sensor ionization chamber current) [4,6]. The impact of such interference has also been taken into account by the authors in the development of databases regarding these operation processes, i.e., use and maintenance. All natural environmental interference directly or indirectly affects the operation process of individual FAS elements, devices or modules [6,53,54]. The ionization detector is particularly susceptible to environmental interference, but, e.g., mechanical oscillations or vibrations impact the functioning of a linear smoke detector [4,17,55,56]. In their FAS operational research, the authors of the paper did not encounter, e.g., general environment measurements in terms of, among others, electromagnetic interference, vibration levels or noise, particularly for systems operated within transport facilities, which require FAS-AWS integration [4,57,58]. Such measurements or observations should already be conducted prior to the FAS design process in order to reduce false alarm probability [4,15,56,58].

Functional reliability under normal conditions, but also in the case of a security threat to the protected facilities, is an important operation-related issue for all ESSs, FASs in particular [4,59,60]. The authors believe that this should be solved in relation to stationary and non-stationary FASs operating at the initial fire stage, as well as during a so-called developed fire within protected facilities [6,61,62]. An FAS is powered by electricity drawn upstream of the main fire switch and should continue functioning for a specified duration, even under conditions of a developed fire [4,6]. Therefore, all technical and organizational solutions increasing the functional reliability of this system under ‘normal’ conditions and upon a fire breaking out should be applied [63,64,65]. Employing redundancy and the fail-safe principle are some of the methods to increase FAS reliability at the system engineering stage [6,66,67].

According to the authors, particular attention should be paid to the process of transmitting alarm and failure signals in the FAS to the ARC and SFB [6,68]. Image transmission from a mobile FAS should also be uninterrupted, and the information on the fire process and its parameters should be continuously forwarded to the command post [16,69,70]. The designer of a given fire device or element (e.g., a detector) already plays an important role in ensuring an appropriate reliability level. These issues must also be considered by the designer of an entire FAS. Such a person takes into account all factors (favourable and unfavourable) affecting an FAS as a whole [4,71]. The so-called site visit prior to the very process of the installation and acceptance testing of an FAS, as well as the determination of a real fire hazard process, are also crucial problems [4,15,65,70].

FAS power supply is a very important FAS operation process. This should be accomplished with voltages exhibiting rated permissible parameters for all elements, devices and modules within detection circuits or loops [4,72,73]. Voltage changes may lead to operational malfunctions of the system, especially in the case of long detection loops and circuits (several hundred m) [6,74,75,76]. A battery bank with a capacity based on the so-called energy balance is used as an FAS backup power supply [6,77,78,79]. Calculating such a balance for an FAS is easy in the case of a focused system. However, this task becomes more complex when dealing with scattered or mixed FASs [15,80,81]. Particular attention should be also paid to the reliability (certainty) and quality of power supply energy for security systems in SCI facilities [4,6,17,82].

An element that determines FCV detection is a detector with a built-in sensor that identifies fire hazards. It is quite different in a non-stationary FAS, where fire hazard detection is based on images or a video transmitted in real time from sensors (cameras) onboard a UAV [4,83,84]. In such a case, the decision on detecting a fire phenomenon can be made on a UAV platform or at an operation (command) post located on a mobile means of transport, see Figure 3 [4,15,17]. The fire phenomenon is then detected using artificial neural networks that continuously classify individual images and detect a threat in the form of smoke, temperature or flame [4,85,86]. Therefore, FAS designers always take into account the type of fuel accumulated in a given room, building or storage, or in the open (e.g., forest). Employing so-called test fires (TF 1–9), they attempt to match an appropriate detector with a sensor exhibiting specific sensitivity [6,87,88]. The task of the detectors is to rapidly respond to a fire phenomenon and generate an alarm signal as soon as possible (change in the technical state—transition from monitoring to alarm) [6,16]. The performance tests conducted by the authors confirmed this construction principle in the case of all FASs [4,6]. Facilities and outdoor areas often experience a change in the manner of applying (using) these planes; however, there are currently no operational recommendations within applicable legislation to take this process, as well as, e.g., detector type change, control matrix organization or FAS functional structure, into account [89,90]. Changes introduced during FAS operation should also take reliability and power supply into account (developing a new energy balance) [6,91,92,93].

An issue that is particularly important in terms of the FAS operation process is a change in the environmental (indoor and outdoor) conditions [4,94,95]. The environment has a significant impact on the technical parameters of detectors, sensors and devices employed for fire detection, leading to variations in detection sensitivity, and distorting characteristics through modifying FCVs, which the alarm state concerns [6,96,97]. Environmental parameters significantly impact the so-called fire triangle [98,99,100]. They affect all FAS technical parameters, e.g., fire phenomenon detection time, false alarm probability, α particle mobility variations in ionization detectors, and FCV changes [4,101,102,103]. Yet another considerable problem associated with the non-stationary FAS operation processes includes selected issues related to tactical and technical parameters, in terms of fire phenomena field detection in particular. The authors of this paper plan to test a demonstrator of such a system and develop articles on its operation process.

## 3. The Most Important Fire Causes and Their Impact on FAS False Alarms

Pursuant to the PKN-CEN/TS 54-14:2020-09 standard [104], FAS false alarms are alarms arising from phenomena different than fire. Examples of such include the following:

- Fire-resembling phenomena (e.g., dust in a room, fog or bonfire smoke);
- Natural environment impact (e.g., steam, fog, rain condensation, hail, etc.);
- Malignant—deliberate fire triggering (so-called sabotage or actions of competitors);
- Alarm initiation in good faith via, e.g., misinterpretation;
- The failure of an FAS, detection loop or circuit monitoring a building or floor;
- The incorrect operation and maintenance of an FAS or FACP.

**Figure 4:**
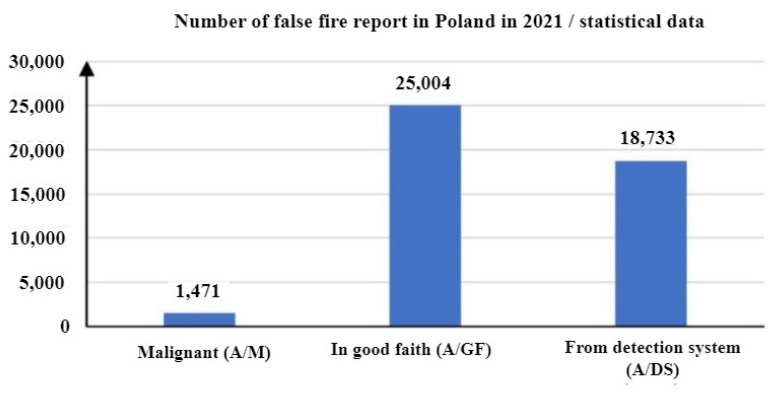
Number of false fire alarms in Poland in 2021.

According to the data of Statistics Poland, the share of false alarms related to FASs amounted to 42.7% of all false fire alarms in Poland in 2021, as illustrated in Figure 4.

**Figure 5:**
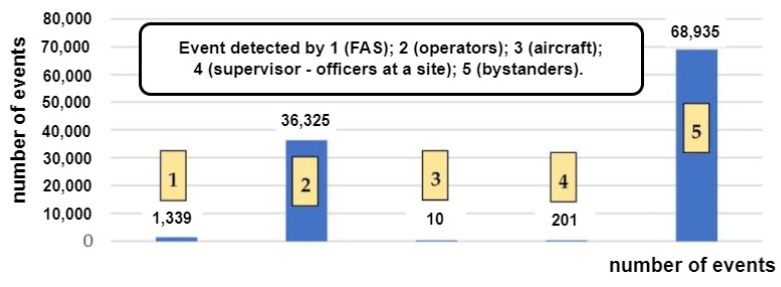
Number of actual fire alarms in Poland, 2021.

**Figure 6:**
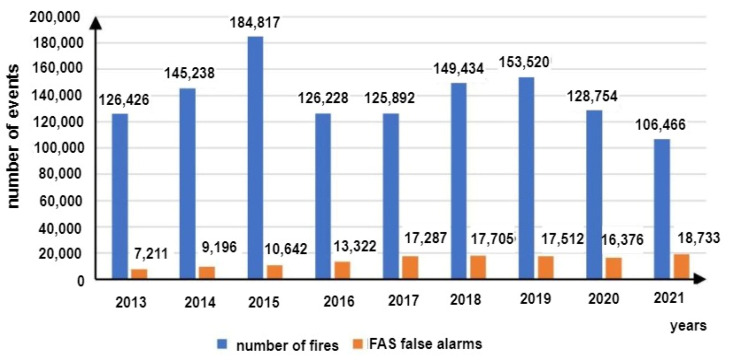
Number of false alarms relative to the number of alarms in Poland.

Statistics Poland has also provided data on real fire alarms, i.e., events that require undertaking actions aimed at restricting fire spread upon FSB arrival at the site. In this case, the share of alarms originating from an FAS amounted to only 1.3% of all real fire alarms, as shown in Figure 5. Figure 6 illustrates the total number of false alarms relative to the number of fires in Poland.

**Figure 7:**
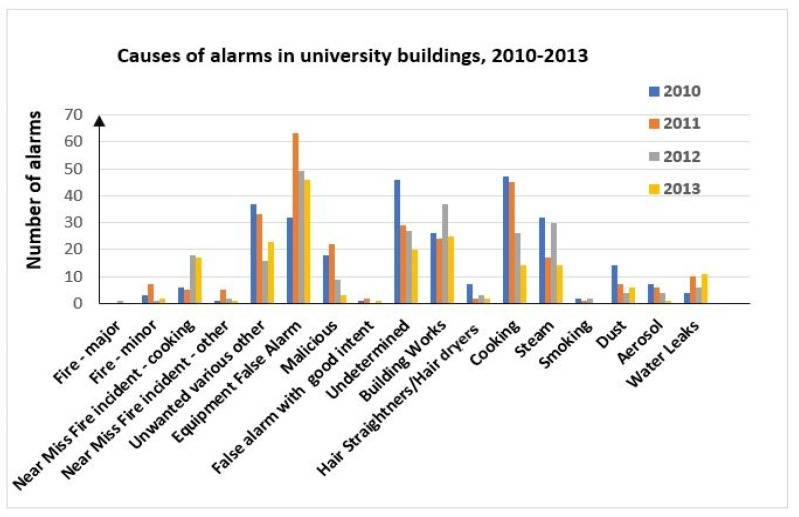
Causes of alarms in KCL university buildings, 2010–2013.

Data regarding these phenomena presented by Statistics Poland for the years 2013–2021 indicate that despite the technical progress in the field of FAS, the share of these systems in the number of false alarms in Poland was increasing. The authors [4,8,105] suggest that this trend is linked with the growing number of buildings, for which an FAS is legally required. The causes behind false alarms in FASs are usually related to partial or complete FAS failure or a phenomenon that exhibits the features of a fire to a certain degree. The authors of [106] reviewed a set of statistical data on fire alarms occurring in the university buildings of the King’s College London (KCL), including false alarms in FASs, as shown in Figure 7.

Phenomena associated with cooking, construction work or failure of the system itself are the primary impact factors for false alarm signals in FASs. In the case of cooking-related false alarms, it can be noted that the majority involve smoke or steam, which may suggest that smoke detectors are more prone to false alarms than other detector types. A significant number of false alarm causes not associated with FAS failures are related to FCVs applied by system detectors to detect fires. The detectors installed within an FAS detect various fire-related FCV. Therefore, other (different) phenomena may trigger false alarms in the case of each of these detectors individually.

Optical smoke detectors operate based on the phenomenon of light absorption or scattering by smoke, which means that each substance or gas that penetrates the inside of the detector may trigger a false alarm. The diffuse optical smoke detector for detecting fires employs electromagnetic waves in the infrared range. A detector chamber contains a transmitter (e.g., LED) and receiver (e.g., photodiode). They are embedded in such a way that the radiation that is constantly emitted by the transmitter is not able to reach the receiver. If the chamber is penetrated by the particles of smoke or any other gas that is able to scatter an infrared beam, a portion of this scattered radiation will be received by the receiver, and thus, the detector will sound an alarm. A linear optical smoke detector also employs infrared. Like the scatter optical detector, a line optical detector is fitted with a transmitter that continuously emits infrared radiation, and a receiver. However, in this case, a receiver in monitoring state receives all of the radiation emitted by the transmitter. If smoke particles appear between the receiver and transmitter, some of the radiation will be attenuated, and thus, less radiation will reach the receiver. In both of these detector types, a false alarm may be triggered by a gas originating from a chemical or physical process, dust, particulate matters, steam and condensation.

A thermal sensor usually uses a thermistor as a detector. Its resistance is a function of the temperature of the material it is made of. A change in the temperature resulting from a fire will lead to a variation on the resistance of this element (thermistor temperature coefficient). There are two different coefficients: negative (NTC) and positive (PTC) temperature coefficients. Resistance changes lead to a varying FCV transducer current, which generates an alarm signal. Heat detectors can be divided into redundant and differential ones. Redundant heat detectors have a trip temperature threshold, while differential detectors respond to the change rate (dynamics) of this parameter. In the case of a heat detector, a false alarm may be triggered by a sudden temperature increase caused by heating equipment, manufacturing processes or device failures.

Flame detectors employ flame electromagnetic radiation. They detect a flame spectrum within a UV range above 300 nm. A detector in such sensors is usually a quartz bulb filled with argon, wherein an incident UV quantum leads to electron knock-out, which combined with a strong electric field causes avalanche multiplication. A current increase over a short period of time indicates an alarm, whereas infrared detectors detect flame electromagnetic waves in the wavelength band of 800–900 nm and more. The radiation reaching the detector passes through an optical filter and reaches a photodetector, which is a photodiode in most cases. A shift register employed in the detector provides additional protection against false alarms. The register is periodically deleted. When the register is overfilled within a pre-set period of time, an alarm signal is generated. These detectors are susceptible to lightning, and a UV lamp and welding, while an IR flame detector is susceptible to very hot objects or modulated sunlight. Limiting the number of false alarms is one of the primary objectives of FAS manufacturers. There are currently numerous publications available that include proposed solutions to certain issues associated with false alarms. The issue of false alarms is crucial (important) when it comes to FAS. The number of these unwanted signals can be limited through, e.g., two-stage alarms (in such a case, a stage 1 alarm requires acknowledgement by a system supervisor) and employing modern detectors (e.g., multisensors—detectors responding to various fire characteristic values, such as smoke, temperature, gas or flame and detectors with memory and employing a trained artificial neural network to track fluctuations, rate or trend of parameter changes). This also involves the application of, e.g., sensor coincidence alarming, changes in alarm signal level in the case of dirty lenses or the employment of pre-clearance and repeated alarm waiting for detectors within two different detection loops. The issue of false FAS alarms has to be thoroughly studied at the engineering stage. This also includes the practical use of premises monitored by the FAS, e.g., welding, artificial lighting sources or the scope of environmental changes.

## 4. Energy Balance Determination Process Graphs for FASs Operating in State Critical Infrastructure Facilities

**Figure 8:**
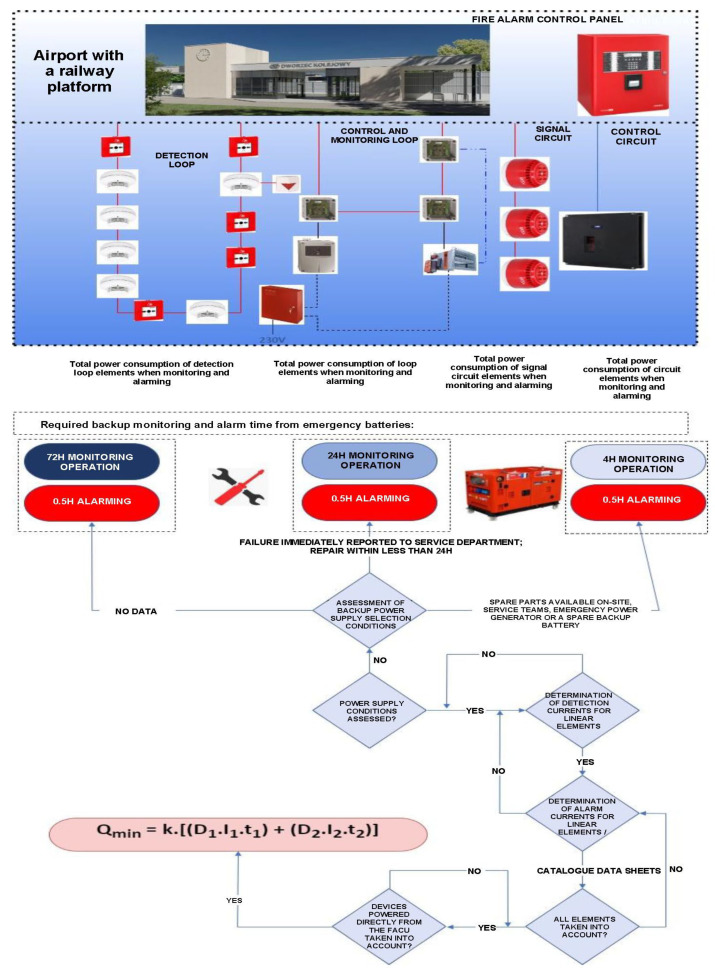
Concentrated FAS energy balance determination graph: k—reserve coefficient, usually taken as 1.25; I_1_—determined FAS power consumption in the technical state of monitoring for the first fire system; t_1_—required operating time of the FAS in the technical state of monitoring; D_1_—coefficient associated with battery capacity upon discharging with the I_1_ current (the value should always be obtained from the battery manufacturer; in practice, 1 is adopted for the FAS); I_2_—determined FAS power consumption in the technical state of alarm; t_2_—required FAS operating time in the technical state of alarm; and D_2_—coefficient associated with reduced battery capacity resulting from drawing large-value power under alarm conditions. In the case of typical FACP operating conditions, the adopted value can be 1; however, in the case of AWS, due to the possible broadcasting of a message over all speaker circuits and to all zones, this coefficient may reach a value up to 1.5, H—hour symbol (H = 60 min).

Due to their internal functional structure and implemented technical features associated with ensuring the fire safety of monitored facilities, FASs can be divided into three groups: focused, scattered and mixed [1,2,92]. Figure 8 shows a concentrated FAS energy balance determination graph. The FAS is used within a vast airport area. It conducts fire surveillance of several small-sized buildings, which is why it exhibits such a technical organization. It has two detection and control loops with connected detectors and control-monitoring devices monitoring the technical state of technical and fire-safety elements integrated with the FAS. The 230 V power supply connected to the FACP is the power source for all elements and instruments along detection loops and circuits. The developed graph takes into account various times of detection operation, from 72, through 24 to 4 h, with separate service team availability variants. The first one is no notification and service, the second is that system unfitness is immediately reported to the service team via an alarm and failure signal transmission device (AFSTD), and the third refers to the service team available at the installed FAS location. In addition, the FAS is equipped with an on-site spare part storage, backup power generator and a battery bank. Pursuant to the applicable legal standard, the permissible alarm time for the energy balance determination cases in question is 0.5 h everywhere.

A focused FAS is supplied with electricity through a separate internal power supply circuit from the central point, i.e., a connection to the building (upstream of the fire switch located outside the protected facility). The main electricity power switch always enables isolating all consumers located within the protected building [95,96,97]. Each FAS separately has its own battery bank monitored by FACP anti-tamper contacts. An energy balance is calculated for a focused FAS structure. It takes into account two technical states, namely, monitoring and alarm. An FAS should operate for a specified time resulting from its application within a vast area. Expressions for calculating the energy balance for such FASs are shown in Figure 8. The k coefficient found in the formula is the so-called reserve coefficient, which expresses redundant battery capacity. It usually takes a value equal to 1.25 [1,95]. An FACP has its own power supply, stabilized and monitored by microprocessor systems in terms of output currents and voltages [97,98,99]. There are additional high-pass filters inside the FAS power supply, which eliminate interference within the power grid, e.g., signal harmonics [95,100,101]. The power supply is also fitted with an overvoltage system [97,102,103]. Grid power supply unfitness is reported to an ARC or service groups as a failure (the system switches to backup power) [97,105,106,107]. Only alarm signals—fire and unfitness—are sent to the SFB via two independent teletransmission channels due to the certainty of the information reaching the recipient [1,108,109,110]. Correct FAS energy balance calculations mean proper functioning during normal and emergency operation. The FAS performance (operational) tests confirmed the occurrence of different errors. They are most frequently made by designers of these systems [1,95,97]. The most frequent mistakes in the course of FAS backup power current balance calculations include the following:

(1) The erroneous reading of current consumption catalogue cards for individual elements and devices in an operating state—monitoring and alarm;
(2) The under- or overstatement of the number of devices hooked up to the FACP itself;
(3) Failing to take all elements hooked up to the FACP itself into account [6,15,111];
(4) Overshooting detection loop or circuit maximum current load;
(5) The under- or overstatement of the required FAS support time during monitoring and alarm states [2,11,69,95];
(6) An excessive capacity of the FACP battery bank (control panel power supply efficiency too low, fails to fully charge this device) [1,18,68,95].

## 5. Operation Process Analysis for a Selected FAS Operating in SCI Facilities

**Figure 9:**
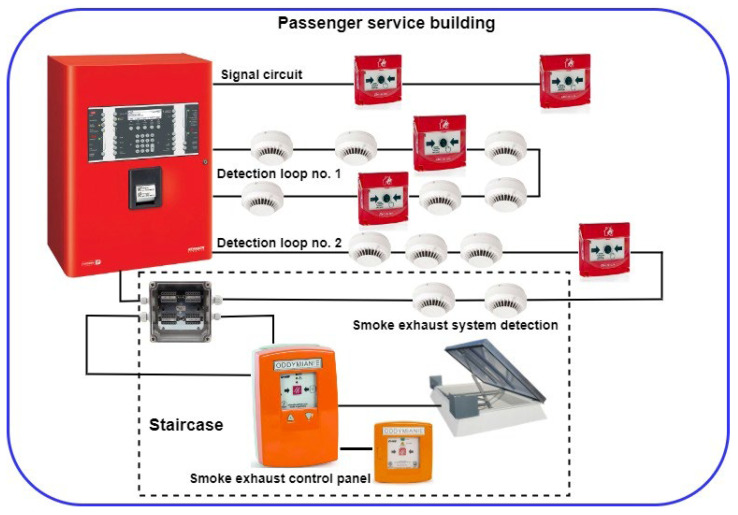
A selected FAS located in an SCI facility, cooperating with an SECP. Fire is detected by a sensor connected to FAS detection loop No. 2—SECP section (zone–group).

In the age of rapid technological progress and constant technical development, buildings and facilities within an SCI area are exposed to numerous different internal and external hazards [1,12,112]. These include fire breaking out that may be an indirect or direct consequence of a terrorist threat. This is why correct protection using active and passive fire safety equipment is a vital issue. The FAS and FED constitute active protections in SCI facilities. Satisfying this requirement determines reliable FAS functioning at all fire stages (initial, in particular) and during the extinguishing operation, i.e., detecting the fire, signalling and triggering FEDs and other fire-fighting systems [1,112,113]. Therefore, appropriately configured and reliable FASs are employed as protection. When operating FAS in SCI facilities and vast areas, a reliable system that guarantees a permissible level of fire safety should be ensured already at the engineering stage. It is of particular importance in the case of SCI facilities. Figure 9 shows an example of an FAS structure operating in an SCI building. The FAS system includes a gravitational smoke exhaust system installed in the staircase. An FAS FACP is coupled with a smoke exhaust control panel (SECP). The SECP is incorporated into an FAS detection loop via an I&C module (I/O). The loop detects FCV phenomena (Figure 9) for the entire staircase located in the SCI building. SECP fire detection is implemented by detectors also located within the FAS detection loop. The detectors were properly assigned to a given detection zone in accordance with the fire scenario and control matrix. SECP activation leads to the triggering of, e.g., a smoke damper, i.e., its opening and the opening of an aeration door at the ground floor of the facility staircase.

**Figure 10:**
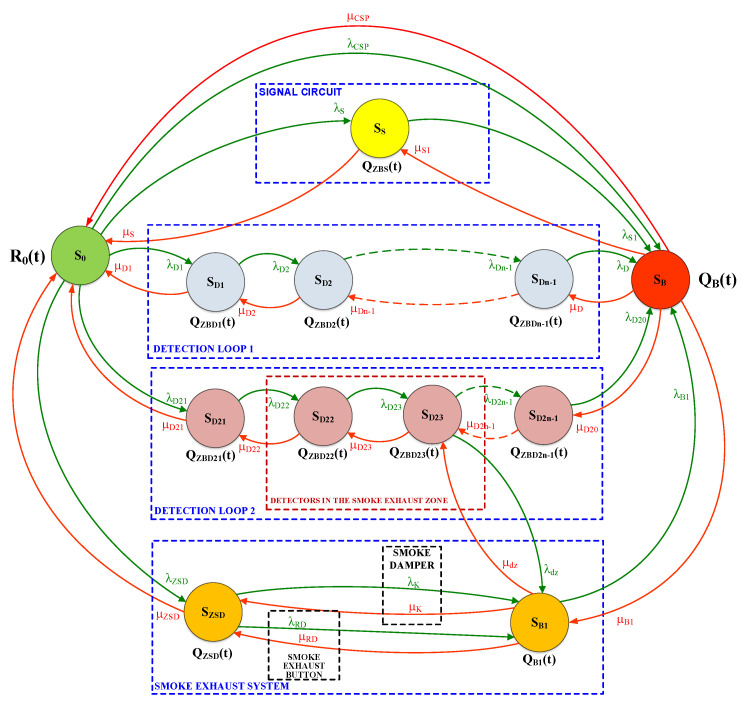
Relationships within a fire alarm system cooperating with an SECP. Fire is detected by detectors connected to the FAS detection loop. Designations in the Figure: R_0_(t)—probability function of an FAS staying in the S_PZ_ state; Q_ZB_(t)—probability function of an FAS staying in the S_ZB_ state; Q_B_(t)—probability function of an FAS staying in the S_B_ state; λ_ZB1_—intensity of transitions from the S_PZ_ state to the S_ZB_ state; μ_PZ1_—intensity of transitions from the S_ZB_ state to the S_PZ_ state; λ_ZB2_—intensity of transitions from the Q_ZB_ state to the Q_B_ state; and μ_PZ_—intensity of transitions from the Q_B_ state to the S_PZ_ state.

By conducting a functional analysis for the FAS, it is possible to illustrate the safety relationships occurring therein in terms of reliability and operation, as presented in Figure 10. The S_0_ state of full fitness is a state in which an FAS correctly implements all of its functions associated with fire detection. The S_ZB_ safety hazard state (e.g., Q_ZBD1_; Q_ZBD2_; …; Q_ZBDn-1_; Q_ZBD21_; Q_ZBD22_; …; Q_ZSD_; Q_ZBS_; Q_ZB1_) is a state in which an FAS partially executes its functions associated with detection, fire scenario implementation and control. The S_B_ safety unreliability state is a state in which an FAS does not fulfil its functions arising from the fire scenario and control—an unfit system. If an FAS remains in the S_0_ state of full fitness and experiences unfitness states of individual devices installed within detection loops or circuits, the system switches to the safety hazard state: S_S_ (signalling circuit), S_D1_, S_D2_, S_D3_, …, (detection loop No. 1), S_D1_, S_D1_, …, (detection loop No. 2) and the smoke exhaust system S_ZSD_, S_B1_. FAS in the state of S_ZB_ safety hazard can switch to the S_0_ state of full fitness upon restoring all relationships and functions implemented by the FAS (recovery process implementation involving the repair and replacement of system devices with an intensity of µ—signalling circuit µ_S1_; detection loop No. 1—µ_D1_, µ_D2_, …; detection loop No. 2—µ_D21_, µ_D22_, …; and the smoke exhaust system µ_K_, µ_B1_, µ_RD_, and µ_ZSD_, respectively). The Q_B_ FAS safety unreliability, in the case of all detection loops and circuits being unfit, leads to the inability to implement functions arising from the fire control matrix. On the other hand, an FAS switches from the S_B_ state of safety unreliability to the S_0_ state of full fitness for FAS when all functions arising from a control matrix are restored. For the sake of better clarity, Figure 10 shows separate transitions resulting from λ damage intensities and µ recovery intensities for individual detection circuits and loops of the selected FAS. Detection loop No. 2 takes into account two detectors functioning within the smoke exhaust zone. The SECP includes mechanical assemblies—a smoke damper with a drive and a separate smoke exhaust button (marked in Figure 9).

In the FAS in question, it is assumed that after the system is commissioned, a facility is fit—implements all assumed functionalities with an R_0_ probability—i.e., it is in the S_0_ technical state. The FAS operation process includes failures (unfitness) leading to partial (S_D1_, S_D2_, …) or complete system failure, the S_B_ state. Partial failures, include, e.g., a power supply, detection loop or detector failure, while a complete—or catastrophic—unfitness means a fire alarm control panel that is not functioning.

An FAS cooperating with SECP as shown in Figure 10 has been described by the following system of the Kolmogorov–Chapman Equation (1):

(1) $${R_{0}}^{\prime }(t)=-\lambda_{CSP}\cdot R_{0}(t)-\lambda_{D1}\cdot R_{0}(t)-\lambda_{D21}\cdot R_{0}(t)-\lambda_{ZSD}\cdot R_{0}(t)+\mu_{CSP}\cdot Q_{B}(t)+\;+\mu_{S}\cdot Q_{ZBS}(t)+\mu_{D1}\cdot Q_{ZBD1}(t)+\mu_{D21}\cdot Q_{ZBD21}(t)+\mu_{ZSD}\cdot Q_{ZSD}(t)\;{Q^{\prime }}_{ZBS}(t)=-\lambda_{S1}\cdot Q_{ZBS}(t)-\mu_{S}\cdot Q_{ZBS}(t)+\lambda_{S}\cdot R_{0}(t)+\mu_{S1}\cdot Q_{B}(t)\;{Q^{\prime }}_{ZBD1}(t)=-\lambda_{D2}\cdot Q_{ZBD1}(t)-\mu_{D1}\cdot Q_{ZBD1}(t)+\lambda_{D1}\cdot R_{0}(t)+\mu_{D2}\cdot Q_{ZBD2}(t)\;{Q^{\prime }}_{ZBD2}(t)=-\lambda_{Dn-1}\cdot Q_{ZBD2}(t)-\mu_{D2}\cdot Q_{ZBD2}(t)+\lambda_{D2}\cdot Q_{ZBD1}(t)+\mu_{Dn-1}\cdot Q_{ZBDn-1}(t)\;{Q^{\prime }}_{ZBDn-1}(t)=-\lambda_{D}\cdot Q_{ZBDn-1}(t)-\mu_{Dn-1}\cdot Q_{ZBDn-1}(t)+\lambda_{Dn-1}\cdot Q_{ZBD2}(t)+\mu_{D}\cdot Q_{B}(t)\;{Q^{\prime }}_{ZBD21}(t)=-\lambda_{D22}\cdot Q_{ZBD21}(t)-\mu_{D21}\cdot Q_{ZBD21}(t)+\lambda_{D21}\cdot R_{0}(t)+\mu_{D22}\cdot Q_{ZBD22}(t)\;{Q^{\prime }}_{ZBD22}(t)=-\lambda_{D23}\cdot Q_{ZBD22}(t)-\mu_{D22}\cdot Q_{ZBD22}(t)+\lambda_{D22}\cdot Q_{ZBD21}(t)+\mu_{D23}\cdot Q_{ZBD23}(t)\;{Q^{\prime }}_{ZBD23}(t)=-\lambda_{D2n-1}\cdot Q_{ZBD23}(t)-\lambda_{dz}\cdot Q_{ZBD23}(t)-\mu_{D23}\cdot Q_{ZBD23}(t)+\;+\lambda_{D23}\cdot Q_{ZBD22}(t)+\mu_{D2n-1}\cdot Q_{ZBD2n-1}(t)+\mu_{dz}\cdot Q_{B1}(t)\;{Q^{\prime }}_{ZSD}(t)=-\lambda_{K}\cdot Q_{ZSD}(t)-\lambda_{RD}\cdot Q_{ZSD}(t)-\mu_{ZSD}\cdot Q_{ZSD}(t)+\lambda_{ZSD}\cdot R_{0}(t)+\;+\mu_{K}\cdot Q_{B1}(t)+\mu_{RD}\cdot Q_{B1}(t)\;{Q^{\prime }}_{B1}(t)=-\lambda_{B1}\cdot Q_{B1}(t)-\mu_{K}\cdot Q_{B1}(t)-\mu_{RD}\cdot Q_{B1}(t)-\mu_{dz}\cdot Q_{B1}(t)+\lambda_{dz}\cdot Q_{ZBD23}(t)+\;+\lambda_{K}\cdot Q_{ZSD}(t)+\lambda_{RD}\cdot Q_{ZSD}(t)+\mu_{B1}\cdot Q_{B}(t)\;{Q^{\prime }}_{B}(t)=-\mu_{CSP}\cdot Q_{B}(t)-\mu_{S1}\cdot Q_{B}(t)-\mu_{D}\cdot Q_{B}(t)-\mu_{D20}\cdot Q_{B}(t)-\mu_{B1}\cdot Q_{B}(t)+\;+\lambda_{CSP}\cdot R_{0}(t)+\lambda_{S1}\cdot R_{0}(t)+\lambda_{D}\cdot Q_{ZBDn-1}(t)+\lambda_{D20}\cdot Q_{ZBD2n-1}(t)+\lambda_{B1}\cdot Q_{B1}(t)$$

If we assume the following baseline conditions for further analysis (2):

(2) $$R_{0}(0)=1,\;Q_{ZBS}(0)=Q_{ZBD1}(0)=Q_{ZBD2}(0)=Q_{ZBDn-1}(0)=Q_{ZBD21}(0)=Q_{ZBD22}(0)=\;=Q_{ZBD23}(0)=Q_{ZBD2n-1}(0)=Q_{ZSD}(0)=Q_{B1}(0)=Q_{B}(0)=0$$

where

*R*_0_(*t*) is the probability function of the system staying in the S_PZ_ state of full fitness;

*Q_ZBS_*(*t*), *Q_ZBD_*_1_(*t*), *Q_ZBD_*_2_(*t*), *Q_ZBDn_*_−1_(*t*), *Q_ZBD_*_21_(*t*), *Q_ZBD_*_22_(*t*), *Q_ZBD_*_23_(*t*), *Q_ZBD_*_2*n*−1_(*t*), *Q_ZSD_*(*t*), *Q_B_*_1_(*t*) are the probability functions for a FAS staying in individual safety hazard states;

*Q_B_*(*t*) is the probability function of the system staying in the S_B_ state of safety unreliability;

*λ_CSP_* is the intensity of transition from the S_PZ_ state of full fitness to the S_B_ state of safety unreliability;

*µ_CSP_* is the intensity of transition from the S_B_ state of safety unreliability to the S_PZ_ state of full fitness;

*λ_S_*_1_, *λ_D_*, …are the intensities of transitions from the S_PZ_ state of full fitness, the S_ZB_ state of safety hazard or the S_ZB_ state of safety unreliability, in accordance with designations in Figure 7;

*µ_S_*_1_, *µ_D_*_2_, …are the intensities of transitions from the S_ZB_ state of safety hazard to the S_PZ_ state of full fitness, and the state of safety unreliability to the state of safety hazard, in accordance with designations in Figure 10.

Then, after applying the Laplace transform to the set of Equation (1), we get a system of linear Equation (3):

(3) $$s\cdot {R_{0}}^{*}(s)-1=-(\lambda_{CSP}+\lambda_{D1}+\lambda_{D21}+\lambda_{ZSD})\cdot {R_{0}}^{*}(s)+\mu_{CSP}\cdot {Q_{B}}^{*}(s)+\;+\mu_{S}\cdot {Q_{ZBS}}^{*}(s)+\mu_{D1}\cdot {Q_{ZBD1}}^{*}(s)+\mu_{D21}\cdot {Q_{ZBD21}}^{*}(s)+\mu_{ZSD}\cdot {Q_{ZSD}}^{*}(s)\;s\cdot {Q_{ZBS}}^{*}(s)=-(\lambda_{S1}+\mu_{S})\cdot {Q_{ZBS}}^{*}(s)+\lambda_{S}\cdot {R_{0}}^{*}(s)+\mu_{S1}\cdot {Q_{B}}^{*}(s)\;s\cdot {Q_{ZBD1}}^{*}(s)=-(\lambda_{D2}+\mu_{D1})\cdot {Q_{ZBD1}}^{*}(s)+\lambda_{D1}\cdot {R_{0}}^{*}(s)+\mu_{D2}\cdot {Q_{ZBD2}}^{*}(s)\;s\cdot {Q_{ZBD2}}^{*}(s)=-(\lambda_{Dn-1}+\mu_{D2})\cdot {Q_{ZBD2}}^{*}(s)+\lambda_{D2}\cdot {Q_{ZBD1}}^{*}(s)+\mu_{Dn-1}\cdot {Q_{ZBDn-1}}^{*}\left(s\right)\;s\cdot {Q_{ZBDn-1}}^{*}(s)=-(\lambda_{D}+\mu_{Dn-1})\cdot {Q_{ZBDn-1}}^{*}(s)+\lambda_{Dn-1}\cdot {Q_{ZBD2}}^{*}(s)+\mu_{D}\cdot {Q_{B}}^{*}(s)\;s\cdot {Q_{ZBD21}}^{*}(s)=-(\lambda_{D22}+\mu_{D21})\cdot {Q_{ZBD21}}^{*}(s)+\lambda_{D21}\cdot {R_{0}}^{*}(s)+\mu_{D22}\cdot {Q_{ZBD22}}^{*}(s)\;s\cdot {Q_{ZBD22}}^{*}(s)=-(\lambda_{D23}+\mu_{D22})\cdot {Q_{ZBD22}}^{*}(s)+\lambda_{D22}\cdot {Q_{ZBD21}}^{*}(s)+\mu_{D23}\cdot {Q_{ZBD23}}^{*}(s)\;s\cdot {Q_{ZBD23}}^{*}(s)=-(\lambda_{D2n-1}+\lambda_{dz}+\mu_{D23})\cdot {Q_{ZBD23}}^{*}(s)+\lambda_{D23}\cdot {Q_{ZBD22}}^{*}(s)+\;+\mu_{D2n-1}\cdot {Q_{ZBD2n-1}}^{*}(s)+\mu_{dz}\cdot {Q_{B1}}^{*}(s)\;s\cdot {Q_{ZSD}}^{*}(s)=-(\lambda_{K}+\lambda_{RD}+\mu_{ZSD})\cdot {Q_{ZSD}}^{*}(s)+\lambda_{ZSD}\cdot {R_{0}}^{*}(s)+\;+\mu_{K}\cdot {Q_{B1}}^{*}(s)+\mu_{RD}\cdot {Q_{B1}}^{*}(s)\;s\cdot {Q_{B1}}^{*}(s)=-(\lambda_{B1}+\mu_{K}+\mu_{RD}+\mu_{dz})\cdot {Q_{B1}}^{*}(s)+\lambda_{dz}\cdot {Q_{ZBD23}}^{*}(s)+\;+\lambda_{K}\cdot {Q_{ZSD}}^{*}(s)+\lambda_{RD}\cdot {Q_{ZSD}}^{*}(s)+\mu_{B1}\cdot {Q_{B}}^{*}(s)\;s\cdot {Q_{B}}^{*}(s)=-(\mu_{CSP}+\mu_{S1}+\mu_{D}+\mu_{D20}+\mu_{B1})\cdot {Q_{B}}^{*}(s)+\lambda_{CSP}\cdot {R_{0}}^{*}(s)+\;+\lambda_{S1}\cdot {R_{0}}^{*}(s)+\lambda_{D}\cdot {Q_{ZBDn-1}}^{*}(s)+\lambda_{D20}\cdot {Q_{ZBD2n-1}}^{*}(s)+\lambda_{B1}\cdot {Q_{B1}}^{*}(s)$$

The BlockSim 2022.0.2 computer application was used to calculate the availability and reliability for an FAS with an SECP staying in individual states, as per the assumed operating time of 8760 h, and the repair (recovery) and reliability coefficient values.

**Figure 11:**
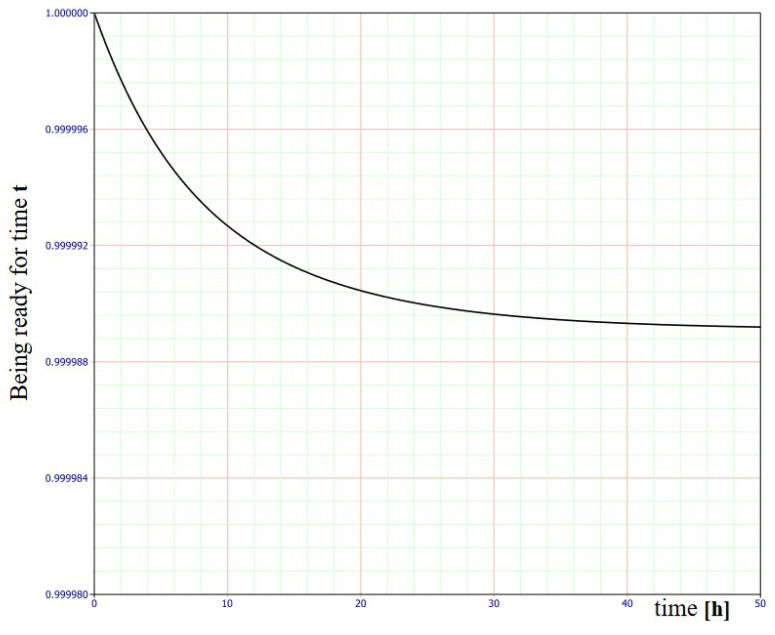
Graphs of availability over time for an FAS in the S_PZ_ state of full fitness, integrated with smoke exhaust and common fire detection.

Figure 11 illustrates the graph of availability over time for an FAS in the S_PZ_ state of full fitness, which is integrated with smoke exhaust and common fire detection.

Table 1 shows only the selected calculations of the FAS availability and reliability coefficients for S_0_, the state of full fitness.

As shown in Figure 9, individual data related to the availability coefficient during the operation of an FAS integrated with an SECP were calculated based on statistical data accumulated from 20 systems operating in various SCI facilities exhibiting a similar functional structure. Determining the λ failure rate and µ recovery rate for individual FAS elements means dividing a facility into sections modelling the entire system, e.g., detectors, modules, signalling devices, SECP, etc. Operation process indicators were determined for these elements. All twenty FASs with an SECP were operated under similar environmental conditions. Based on the presented relationships and calculations conducted using a computer application, it can be noted that a previously designed and fabricated FAS with SECP exhibits a very high availability coefficient k_g_(t). For time t = 1.125 [h], k_g_(t) = 0.999998632 (Table 1). Individual devices making up an FAS with SECP are based on appropriate technical and system solutions. They employ redundancy, and the so-called fail-safe principle. A significant issue associated with the FAS operation process involves the so-called ‘infancy failures (unfitness states)’. The FAS reliability tests conducted by the authors demonstrate the application of appropriate electronic devices, i.e., system components that exhibit high functional reliability. Therefore, if an FAS is treated as a complex technical security system, it also comes with reliability, which is virtually equal to unity at the initial operation process stage. These technical studies, conducted and discussed as part of the paper, confirm the viability of employing various techniques to increase FAS reliability at the engineering stage, e.g., redundancy or safe failure.

## 6. Primary Technical and Tactical Assumptions Related to Non-Stationary FAS Fire Detection Systems within a Vast Area

The authors also study FASs (mobile) located onboard a UAV platform. Chapter five of this paper reviews basic requirements, as well as technical and tactical assumptions for such a security system. These are just preliminary assumptions that will undergo changes in the course of practical tests using a UTV, and a system for processing visual data streams, as well as a neural network implemented onboard the drone [108].

**Figure 12:**
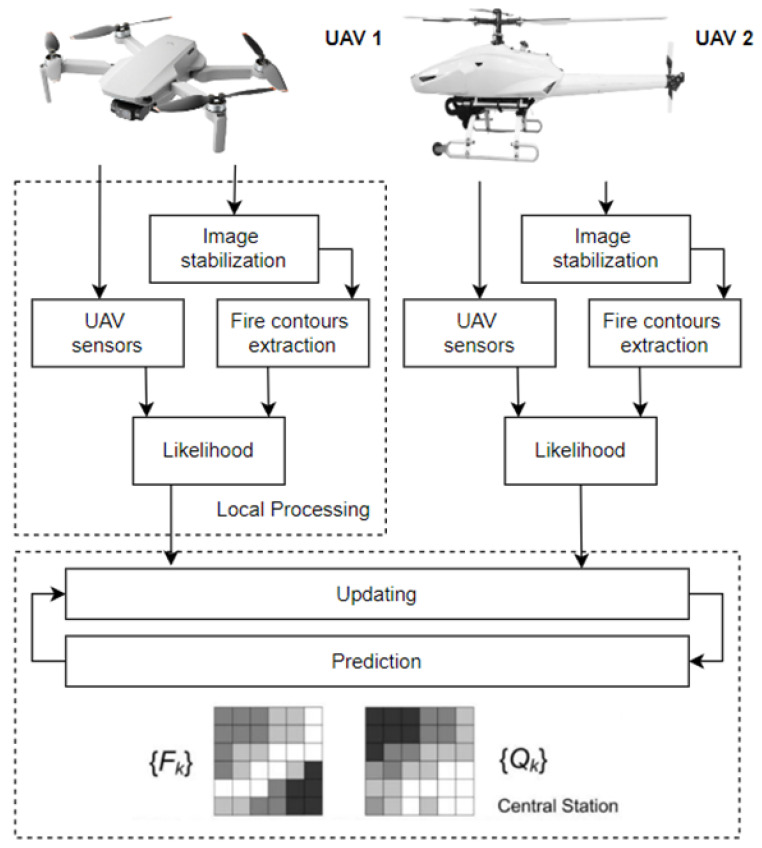
Functioning of a fire reconnaissance system, estimating two available probability ‘grids’ (presence of flame (fire) and fuel in each elementary cell). A dynamic, variable grid state (covering a specified area) is estimated based on the UAV fleet in-flight data. The next stage involves predicting, i.e., taking into account the increase/decrease in uncertainties related to fire movement (direction) within a vast area.

The majority of traditional methods for detecting fires of forests (vast areas) are based on detecting hazardous phenomena with the help of human patrols, smoke detectors, thermal detectors, and watchtowers equipped with optoelectronic cameras. They also involve the application of satellite images and patrol data provided by conventional aircraft patrols. The principle of the operation of smoke and thermal detectors is point-based (they cover a small detection area expressed in m^3^). Their use is unable to reliably provide information on the location and magnitude of a fire within a vast area. This is also elevated by the issue of wireless notifications—the field propagation of electromagnetic fields. Human patrols are characterized by a limited field of view, required shifts due to human organism regeneration and a high percentage of false alarms and involve detection with the use of sight additionally armed with, e.g., binoculars or a camera with an imaging panel [109,110]. Other methods for detecting fires over vast areas include the use of satellite images. Satellites provide complete area coverage; however, the image resolution and potential observation of fire hazards in real time are troublesome, hence making this system less effective due to information processing duration. This includes signal source, ground receiver, forwarding the signal to the SFB, further information and only then undertaking actions locally. The popularity of unmanned aerial vehicles (UAVs) results in their functionality having numerous applications associated with observation and monitoring. While traditional aircraft can be an effective system for detecting fire scenarios, they entail a number of hazards and operating costs, e.g., flight technical safety. Employing aircraft for fire detection generates considerable costs related to purchasing often expensive aerial vehicles. This also includes staff training, and thus, exposing human life to direct danger, particularly during flights at low altitudes and in smoke (imaging accuracy) [108,111]. Approximately 80 pilots have been killed during fire-fighting operations in the USA over the last 20 years [109,110]. In opposition to the traditional systems, when designing modern solutions, engineers turn to autonomous drones. UAVs (unmanned aerial vehicles) are automatic systems that can receive information from high-risk zones. They often provide a view of places hard-to-access by conventional machines due to, e.g., manoeuvrability, flight speed or mobility. They can also conduct missions at night, without risking human life due to flying under difficult conditions. When on a mission, UAVs guarantee real-time transmission, providing SFB fire-fighters with information required to undertake and supervise fire-fighting operations. This entails a number of benefits associated with, e.g., UAV flight dynamics, price and adaptability. Therefore, these objects have become one of the most important elements in detecting fires over vast areas. The selection of detectors and algorithms, as well as the type and quantity of aircraft, leads to the development of new technologies and different approaches to the reconnaissance of a widespread fire phenomenon. The first one described is a perceptual system for monitoring forest fires using a UAS (unmanned aerial system) [108,110]. Its operation takes into account all information collected by an entire UAV fleet to estimate fire expansion (spread rate) using data fusion techniques, see Figure 12. Each of the vehicles processes recorded images only locally, with the data then sent to a central station, where basic fire indicators are estimated [112,113]. The basis for system operation includes the segmentation of the image projected from the camera onto a grid of rectangles, wherein the state of each cell k is determined via two binary values F_k,t_ {1,0}. This is followed by an indication whether there is fire in a given cell, and Q_t,t_ {0,1}, which indicates if combustion products (fuel) are completely depleted. For each primary cell, the system stores two probability values f_k_ = p (F_k_,_t_ = 1)—a fire present in a given cell, and q_k_ = p(Q_k_,_t_ = 1)—fuel in this cell completely depleted. In addition, each of the elementary cells divided this way stores information on the 3D position p_k_, determined by a pre-loaded digital map of the vast area, with particular reference to the altitude over the observed terrain [109,114]. The presented fire spread prediction model is very simple and takes temporal and spatial relationships between individual cells into account. Its main objective is, on the one hand, to incorporate a certain memory type into the estimation process, and, on the other, to make sure that the fire does not spread ‘backwards’, through previously visited zones within a vast area [3,110,115]. Spatial prediction of the received information is also conducted in real time. This is executed to smooth out the so-called estimated fire front evolution within a vast area, e.g., the real-time determination of hazard spread direction. Temporal transition probability for each cell can be distributed in time and expressed as (4).

(4) $$p\left(F_{k},t/F_{k},\;t\;1\right)=Q_{k},\;t-1\;\;\;p(F_{k},t/F_{k},\;t\;1,\;Q_{k},\;t\;1\;p\left(Q_{k},\;t\;1\right))\;p\left(Q_{k},t/Q_{k},\;t\;1\right)=F_{k},\;t-1\;\;\;p(Q_{k},t/F_{k},\;t\;\;1,\;Q_{k},\;t\;\;\;1\;p\left(F_{k},\;t\;1\right))$$

**Figure 13:**
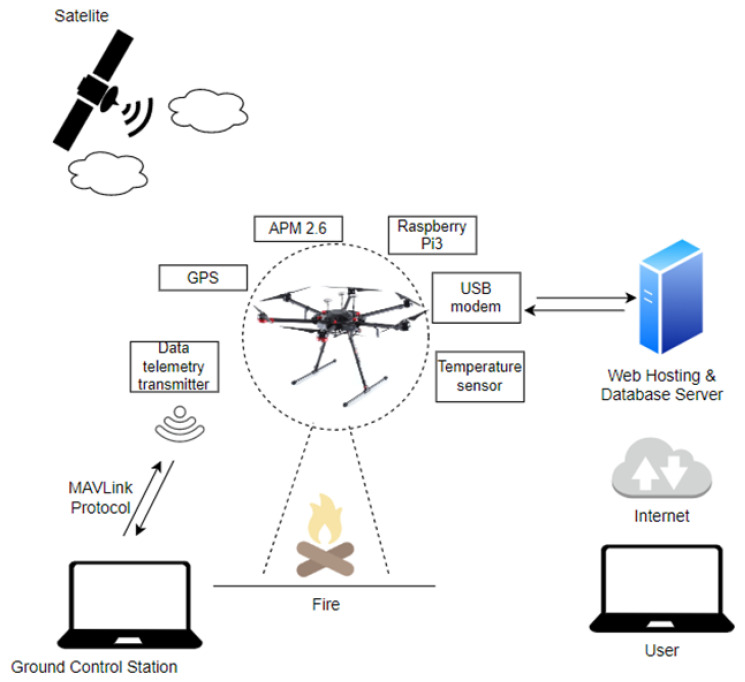
Fire surveillance and monitoring system configuration; own study.

A camera employed as part of this solution is a common (it does not require advanced technologies and processing) infrared camera. It does not provide temperature measurement, but develops, e.g., radiation intensity ‘estimation’ for the entire recorded frame (recorded image). The obtained measurement result thresholds are then proposed for fire spread segmentation within a given area. The next stage of the processing algorithm operation involves proposing electromagnetic radiation intensity thresholds within this spectral range that can be applied for the segmentation of this fire phenomenon (thresholds can be dynamically changed by the system or manually by operators participating in the fire-fighting process). The adopted solution involves thresholding methods based on prior training using artificial neural networks. The training stage always requires selecting training images (fires of different, e.g., intensity, magnitude, smoke volume, etc. in the frames) and corresponding desired threshold values determined by an experienced user. The learning stage always includes determining baseline conditions, where pixels should be deemed as belonging to a given object of interest; the fire source in this case [110,116]. The preliminary stage provides a so-called outline of segmented areas from a given fire hazard. However, this outline is additionally characterized by peculiar parameters to distinguish between the pixels of the outline associated with the so-called fire front. This also involves determining pixels related to the upper part of the flames in a given fire. This provides flame height, always expressed in pixel coordinates. Fires are always characterized by dynamic properties of the flame base and flames within the hazard spreading over a vast area. The position of the fire baseline pixels on the camera image plane (imaging panel) always changes slower than the flame pixel position, which is a phenomenon dynamic over the imaging duration (e.g., fire flame flicker measure is a range of low frequencies) [8,21,110,117]. Another approach towards fire prediction and detection involves employing a non-contact infrared sensor onboard a UAV. In such a case, the UAV equipped with a so-called Ardu Pilot flies autonomously following a pre-set route within the vast area [108,110]. Communication in such solutions is guaranteed by a wireless data link, with the frequency most usually being 433 MHz due to electromagnetic wave propagation at very low altitudes, taking into account natural obstacle attenuation, e.g., trees [4,118,119]. The Raspberry Pi onboard a UAV platform then sends telemetry data to an Internet server. The data is later available online, on a website accessible to operators participating in the fire-fighting operation. The developed system is able to detect forest fires from a minimum altitude of up to 20 m, and at a constant flight speed as low as 5 m/s. This solution is characterized by high simplicity and low prime costs. Unfortunately, employing only a temperature sensor is not technically effective in terms of generating a fire hazard signal, but may constitute a functioning, basic mobile FAS. Justifying a fire phenomenon detection based solely on ground surface temperature data is perfectly fine; however, it is insufficient in terms of the dynamic process of extinguishing fires over a vast area, especially in the case of a so-called ‘firestorm’ phenomenon. In this case, a ‘trip’ threshold for a given FAS temperature predefined by an operator or a neural network system may be very troublesome in terms of application within the detection process, particularly at early fire stages—low values of all FCVs (sides) of the so-called fire triangle components (temperature, fuel and oxygen) [6,16,120,121]. This technical solution of a mobile FAS is illustrated in Figure 13. Mobile fire monitoring uses all available wireless communication technologies and a local, UAV-based system for the pre-processing of a sequence of dynamic images varying over time; this means UAV flight and the changing process, or the fire condition already on the ground plane—altitude, area, smoke intensity, etc. [109,111,117].

**Figure 14:**
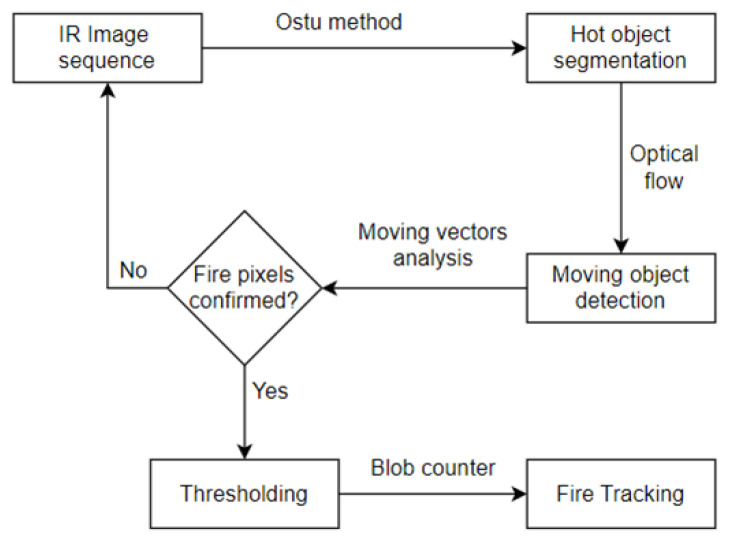
Fire detection algorithm using a mobile FAS; own study.

A number of different approaches have been developed for stationary forest fire detection using local infrared range cameras; however, a small number of these have been adapted to be carried by unmanned mobile, aerial vehicles [122,123]. One of the proposed fire phenomenon detection methods employing infrared techniques is the so-called hot object segmentation (fire locations within strictly defined planes within the area), while simultaneously detecting and predicting spread motion, direction within a given, hard-to-access area (e.g., mountains, swamps). A simplified principle of the operation of such a mobile system can be described in a few steps, as an algorithm illustrates in Figure 14. The first stage is always detecting the so-called hot objects (areas) as potential fire sources, using the so-called histogram-based segmentation method. This is followed by a processing step applying the classic so-called optical flow method for detecting mobile objects in order to eliminate stationary elements that are not fire. This method is often applied in radar stations for the so-called fixed echo suppression, e.g., rainfall (R), hail (H), snow (S), etc. These systems use the difference in the speed of the aircraft and interference (R, H and S) within the observed space. In such a case, motion vectors are always calculated based on the previous (prior) stage, and are analysed to reduce the rate of false fire alarms triggered by moving hot objects. This is a comparison of earlier recorded images in the memory with current ones, and the isolation of significant changes within a given, defined, short period of time. The ultimate stage in the operating sequence of this algorithm is the so-called tracking via a pattern (processing system) of fields containing the detected fire phenomenon and its prediction over time under the impact of changing environmental conditions, e.g., wind speed, precipitation, temperature, humidity, etc.

Most infrared cameras only measure heat distribution and develop single-channel images. Fire pixels within the received camera image are always high-intensity areas. Their local brightness maximum values are a dominant clue in terms of fire pixel classification. The segmentation stage is based on the Otsu method, which is adapted to the automatic thresholding of dynamic images based on the so-called histogram. This method assumes that images for processing always have only two pixel types, namely, foreground and so-called background. The optimum threshold distinguishing between these two classes is always calculated iteratively. Their total spread (intra-class variations) should be maximized. Yet another stage, already discussed previously, is detecting field movements of a fire using the so-called optical flow. Air flow (movement) within a given area always causes fire and flame motion. Fire (flame) motion function is widely applied within the so-called visual fire detection to improve fire detection effectiveness, e.g., reduced false alarm rate. A usually bright moving area is marked as a potential fire spot within a vast area, on an image captured by an infrared camera. However, detecting a fire based solely on these parameters, i.e., motion and brightness within the obtained image, leads to many so-called false alarms (FA). The undesirable FA parameter may be due to the appearance of such hot objects within the observed field as, e.g., vehicles, people and animals [108,111]. In addition to detecting features and motion, the proposed solution involves analysing their changes within the optical flow field to differentiate fires from other hot objects moving in a given space. Optical flow that plays a crucial role in motion detection and analysis for the purposes of processing by computer systems (stationary—on the ground or onboard a UAV), is also employed as part of tests related to fire detection. It is generally described as a two-dimensional distribution of brightness pattern movement speed in images obtained during optical observation with cameras. In such a case, one pixel in an image corresponds to one velocity vector, which creates an optical flow field that can be employed for a more advanced analysis, e.g., in systems supporting fire detection, which utilize an artificial neural network [108,120]. The expression, image brightness variability function can then be written as the relationship [5].

(5) $$\frac{d}{dt}I=\frac{\partial I}{\partial x}u+\frac{\partial I}{\partial y}\nu +\frac{\partial I}{\partial t}=I_{x}u+I_{y}\nu +I_{t}=0$$

where vector *I*(*x*, *y*, *t*) represents the brightness function for images including coordinate (*x*, *y*) parameters within the observed space and over a given time *t*. The flow vector represented by (*u*, *v*) is the velocity of a pixel moving over a vast space (*x*, *y*), where a fire hazard is likely to occur.

**Figure 15:**
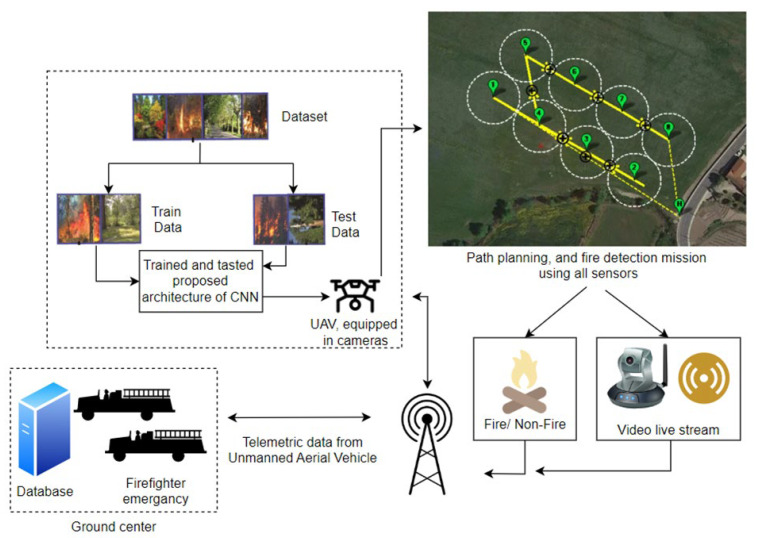
Architecture of the proposed system for detecting and observing fire phenomena using an unmanned aerial vehicle.

The signal processing automation process, artificial intelligence and machine learning processes (neural networks) are applied as part of numerous technical solutions, including for detecting fires over vast areas. This solution has already been employed in practice to scan licence plates, detect facial features in biometric systems and, e.g., categorize one’s own photos on a smartphone. This is only a fraction of the exemplary applications of artificial neural networks. However, neural networks are part of the machine learning function and constitute so-called deep learning algorithm fundamentals. The method for detecting fires over a vast area proposed by the authors uses the so-called convolutional neural network. The equipment of a conceptual fire phenomenon detection system includes an unmanned aerial vehicle with a flight route planning and management system, a Raspberry Pi minicomputer, a communication module, an infrared and visible band camera, and a ground signal station (GCS). The first stage in the design of such a system is the implementation of a neural network, thus, the selection of appropriate architecture. There are numerous possibilities when it comes to selecting suitable neural network architecture. Examples include MobilNetV1, ResNet, AlexNet, VGG and many more. The main issue that is inherent to preliminary testing having access to a required set (quantity) of available images for training neural networks. Fire detection using an unmanned aerial vehicle is an additional complication. Networks have to be trained for photos taken from a given perspective, e.g., bird’s eye view. Modern photographic databases contain images useful in training such a model. Examples include a database of images collected during the forest fires in Arizona, USA [107,109]. However, it should be borne in mind that these sets are very limited. The problems associated with preparing a suitable image database have been previously addressed in numerous research papers worldwide. Many of them discuss various techniques for augmenting such data, based on, e.g., image vectoral conversions, using green screens, etc. Fire phenomena detection within the proposed solution will be based on the data ‘collected’ from two cameras. The application of a duplex system and two functionally independent neural networks will contribute to minimizing the FA index. The fire scale and spread direction within a vast area are important from the perspective of rescue teams participating in the fire-fighting operation. Many of the commonly available UAVs offer only wind strength and direction measurements [108,110,111]. Fusing data from the camera, and built-in accelerometer as well as gyroscope may provide additional information on the direction of, e.g., fire spread and its extent. The communication module fitted on the UAV sends telemetry information to a ground station in real time. The station is a node between the mobile platform and authorities undertaking fire-fighting operations—the SFB and the Voluntary Fire Brigade (VFB). The UAV is the primary element for detecting and notifying the SFB about a fire event. In addition, in the further course of the fire-fighting operation (variable and increasing operational time interval), it provides instantaneous visual information regarding fire development in a given area. Modern autopilot systems implemented onboard commercial UAVs also offer numerous additional functions. A mobile FAS will then become more autonomous. The first of such functions can be the so-called waypoint flight. The UAV receives preset navigational coordinates that constitute flight coordinates for such a machine. In such a scenario, it is possible to imagine that a fire-fighter or any other supervising person enters the area to be monitored by the UAV, whereas the fire detection process becomes fully autonomous. Yet another functionality of the automated control system can be returning to a given take-off point where the operator is located. An unmanned aerial vehicle, after using up a significant portion of its battery capacity, autonomously returns to the take-off point, where it can spontaneously hook-up to a power charging point. The task of the first drone is then taken over by another UAV waiting for its turn to conduct area surveillance. The described functionality of a conceptual system is, in theory, effective and competitive to other, different fire detection methods. However, please note that a software implementation of such a solution is not easy, especially with the data processing and real-time data transmission. The limitations imposed by UAV payload capacity, mobile CPU speed, weather conditions and image database availability are currently the main problems for many engineers designing mobile FASs [107,108,110]. Figure 15 illustrates an architecture example for the proposed fire phenomena detection and observation system using UAVs, developed by the authors of this research paper. Other very important operational aspects related to a UAV detecting fire phenomena over a vast area include flight time and battery bank charging time, and thus, the weight of sensors, as well as electronic and telecommunications modules fitted onboard the UAV. These are only some of the relevant tactical UAV parameters, in addition to, e.g., speed, manoeuvrability within flight planes, climb rate, weight and mass. Preparations for returning to flight should be as minimal as possible. This includes the shortest possible battery bank charging time or spare battery banks, which should be located at the UAV landing site.

## 7. Conclusions

The issues related to the operation of fire alarm systems located in critical infrastructure facilities are extremely important with respect to ensuring their operational safety. These systems are installed and operated in accordance with applicable standards and regulations, as well as legislation, which require FAS monitoring in strictly defined buildings. Such legal empowerment relative to FAS provides high reliability of these systems and an advantage in relation to other security systems. FAS installed in critical infrastructure facilities require the continuous supervision of service teams and the existence of the so-called “on-site spare part storages” to ensure the shortest possible repair (recovery) time. Such a technical solution associated with the operation process means that the recovery rate µ is very low. This means that the repair time of an unfitness is very short. In the case of the presented FAS, the repair (recovery) time during the research conducted by the authors of the article varied, e.g., FAS basic industrial mains power supply failure resulted in a maximum repair time of 15 min. Nevertheless, there were also FAS unfitness events when the repair time took hours, e.g., repairing the unfitness of a detector in the detection loop No. 2/55, reported to the FACU as a “not responding—communication error” malfunction, took 4 [h]; replacing batteries with new ones on the backup power supply takes 13 h 10 min; and the communication (signal transmission) error in loop No. 1 involves a repair time of 4 [h], involving an appropriate tie-in of loop No. 1 cabling to FACP terminals (all unfitness states are messages on an FACP LCD panel). In the process of operating an FAS in SCI facilities, sometimes the times associated with repair/recovery are acceptable and sometimes they are not, due to being longer. In such a case, the entire FAS system or a part of it fails to monitor fire safety within a facility. FAS, and especially an FACP that is responsible for implementing the process of diagnosing systems, is fitted with an extensive module(s) intended solely for this type of operation, so as not to overload the main processor. The FAS diagnosis process is implemented continuously by the FACP, concurrently with the fundamental operation type, i.e., monitoring. However, an alarm signal has higher priority in the FACP, and in the event of any such signal, the control unit automatically suspends the FAS diagnosis process. The issue of FAS energy balance calculations, with its process graph shown in the paper, should always take into account the functional structure of these systems. Developing an operation process model for a selected FAS operating within SCI facilities enabled the calculation of reliability ratios. Based on the conducted computer simulation, the FAS was characterized by a high reliability level in the order of R = 0.988581951 after one year of operation under the given variable environmental conditions. No complete, critical FAS failure was identified based on the conducted actual operational tests and readings taken from the “event log” stored in the FAS non-volatile memory. Due to the functioning of the system, FAS reliability structures do not include so-called “critical paths”. In such a case, the failure of one component or device only leads to the total unfitness of the entire system. An FACP is an element critical for all currently operating FASs for reliability reasons. However, all FACPs in SCI facilities always exhibited 100% redundancy. Then, the second alarm control panel operated as a ‘hot’ unloaded backup. No case of a complete FACP failure was identified in the course of the tests. The conducted tests provide users and persons employed in FAS service departments with current, permanent and actual information on system reliability. In addition to technical aspects, such a high FAS reliability is also important from the legal perspective of the operation process. FASs are the only electronic security systems whose function in buildings is legally assented. The conducted computer simulations also confirm an important practical aspect of the research conducted and scientific considerations discussed herein. Appropriate technical service organization, the functioning of the spare part storage within a given facility, adequate failure response time, an ongoing and rapid diagnostic process and the application of all available technical solutions with respect to improving reliability mean achieving success in the form of a high system availability coefficient. In future research, the authors plan to execute operational tests of FASs operating in other facilities, facilities not classified as SCI. The authors also plan to conduct operational tests involving other electronic security systems operating in so-called smart buildings and single-family houses. In the last chapter, the authors presented initial tactical and technical assumptions for a mobile FAS located on a UAV. They discussed the requirements and methods for processing signals received from UAV-mounted sensors that would change due to the conducted tests. These include preliminary research focusing on constructing a mobile UAV enabling fire reconnaissance and the prediction of a fire hazard over a vast area as a function of time, e.g., forest, mountains and hardly accessible land, such as swamps. When operating an FAS in a building, a user should pay attention to a number of technical and tactical parameters exhibited by such a system operating in an adverse environment, e.g., functional reliability, aspects of power supply involving backup sources, the number of false alarms within a given operation year, etc. The FAS user should particularly focus on a change in the manner of premise use, e.g., the accumulation of large volumes of ‘fuel—wood, furniture, etc.’ and also to the expansion or additional subdivision of building spaces. This should be reflected in changes to a previous FAS design, the type or number of detectors, and, under specific technical conditions, the correction of fire zones within a facility.

## Figures and Tables

**Table 1:** Calculations of the FAS availability and reliability coefficients for the S_0_ state.

Computation Step	Time [h]	Availability (t)	Reliability (t)
0	0	0	0
1	1.125	0.999998632	0.999998525
2	2.125	0.999997576	0.999997214
…	…	…	…
8758	8758.125	0.999989118	0.988584381
8759	8759.125	0.999989118	0.988583085
8760	8760	0.999989118	0.988581951

## Data Availability

The data presented in this study are available on request from the corresponding author.

## Symbols and Acronyms

FAS	fire alarm system
FED	fixed extinguishing devices
ESS	electronic security system
AWS	audio warning system
MCP	manual call point
k_g_(t)	availability coefficient
µ	recovery rate coefficient
λ	failure rate coefficient
R_O_(t)	probability function of an FAS staying in the S_PZ_ state (full fitness)
Q_ZB_(t)	probability function of an FAS staying in the S_ZB_ state (safety hazard)
Q_B_(t)	probability function of an FAS staying in the S_PZ_ state (safety unreliability)
λ_ZB1_	failure rate, transition of a selected FAS from the S_PZ_ state to the S_ZB_ state
μ_PZ1_	recovery rate, transition from the S_ZB_ state to the S_PZ_ state
TF 1–TF 9	test fire designations
AFSTD	alarm and failure signal transmitting device
P_D1_	detection loop No. 1
λ_CSP_	intensity of transition from the S_PZ_ state of full fitness to the SB state of safety unreliability
